# The tumor microenvironment of medulloblastoma: from emerging biological insights to novel therapeutic targeting

**DOI:** 10.3389/fonc.2026.1812994

**Published:** 2026-08-18

**Authors:** Franklin Chien, Marina Michaud, Martha Stewart, Sheila McThenia, Leon McSwain, Andrey Tikunov, Matthew J. Schniederjan, Todd Golde, Tim Gershon, Manoj Bhasin, Tobey J. MacDonald

**Affiliations:** 1 Department of Pediatrics, Emory University School of Medicine, Atlanta, GA, United States; 2 Children’s Healthcare of Atlanta, Aflac Cancer and Blood Disorder Center, Atlanta, GA, United States; 3 Department of Pathology and Laboratory Medicine, Emory University School of Medicine, Atlanta, GA, United States; 4 Department of Pharmacology and Chemical Biology, Emory Center for Neurodegenerative Disease, Emory University, Atlanta, GA, United States; 5 Department of Neurology, Emory Center for Neurodegenerative Disease, Emory University, Atlanta, GA, United States; 6 Department of Biomedical Bioinformatics, Emory University School of Medicine, Atlanta, GA, United States

**Keywords:** medulloblastoma, microenvironment, single-cell, spatial, tumorigenesis

## Abstract

Medulloblastoma is the most common pediatric malignancy of the brain and spine and a major cause of childhood cancer-related morbidity and mortality. Novel therapeutic approaches are urgently needed. The malignant behavior of this disease is influenced both by cell intrinsic factors and by the complex interactions of the tumor cells with its tumor microenvironment. Recent scientific and technological advancements allow increased ability to interrogate the microenvironment in terms of composition and cellular and signaling crosstalk, which together are critical for the regulation of tumor survival, disease proliferation, metastasis, and response to treatment. We summarize the current understanding of the medulloblastoma tumor microenvironment and explore its influence on disease pathogenesis, as well as identify emerging opportunities to leverage the microenvironment as a novel therapeutic target.

## 1 Introduction

Medulloblastoma (MB) is an embryonal tumor of the cerebellum (1). It is the most common childhood cancer of the central nervous system (CNS), and as such, constitutes a significant contributor to pediatric cancer-related mortality. For non-infant patients, treatment involves an intensive multimodal approach, including maximal safe resection, craniospinal irradiation (CSI), and successive cycles of multiagent chemotherapy (2). Patients considered too young for radiation therapy instead receive myeloablative or myelosuppressive high-dose chemotherapy often with autologous stem cell transplant. Survivors frequently experience significant, longstanding neurocognitive deficits as a result of the therapy received (2, 3).

Four molecular subgroups have been identified in MB: Wingless/Int-1 (WNT), Sonic-Hedgehog (SHH), Group 3 (G3), and Group 4 (G4) (4, 5). Molecular subtyping has prognostic implications, with WNT MB having superior outcomes compared to G3, which has the poorest outcomes.

Many patients do not survive their disease despite molecular risk stratification and intensive multimodal therapy. Mortality rates are even higher for those with high-risk features such as the presence of metastasis at diagnosis (M+ staging), inability to achieve gross total resection, or unfavorable molecular markers (6). In the relapsed setting, there are few curative regimens (7). This, in concurrence with the significant neuro-cognitive sequelae, reveals a critical need for novel and more targeted therapy.

**Figure 1:**
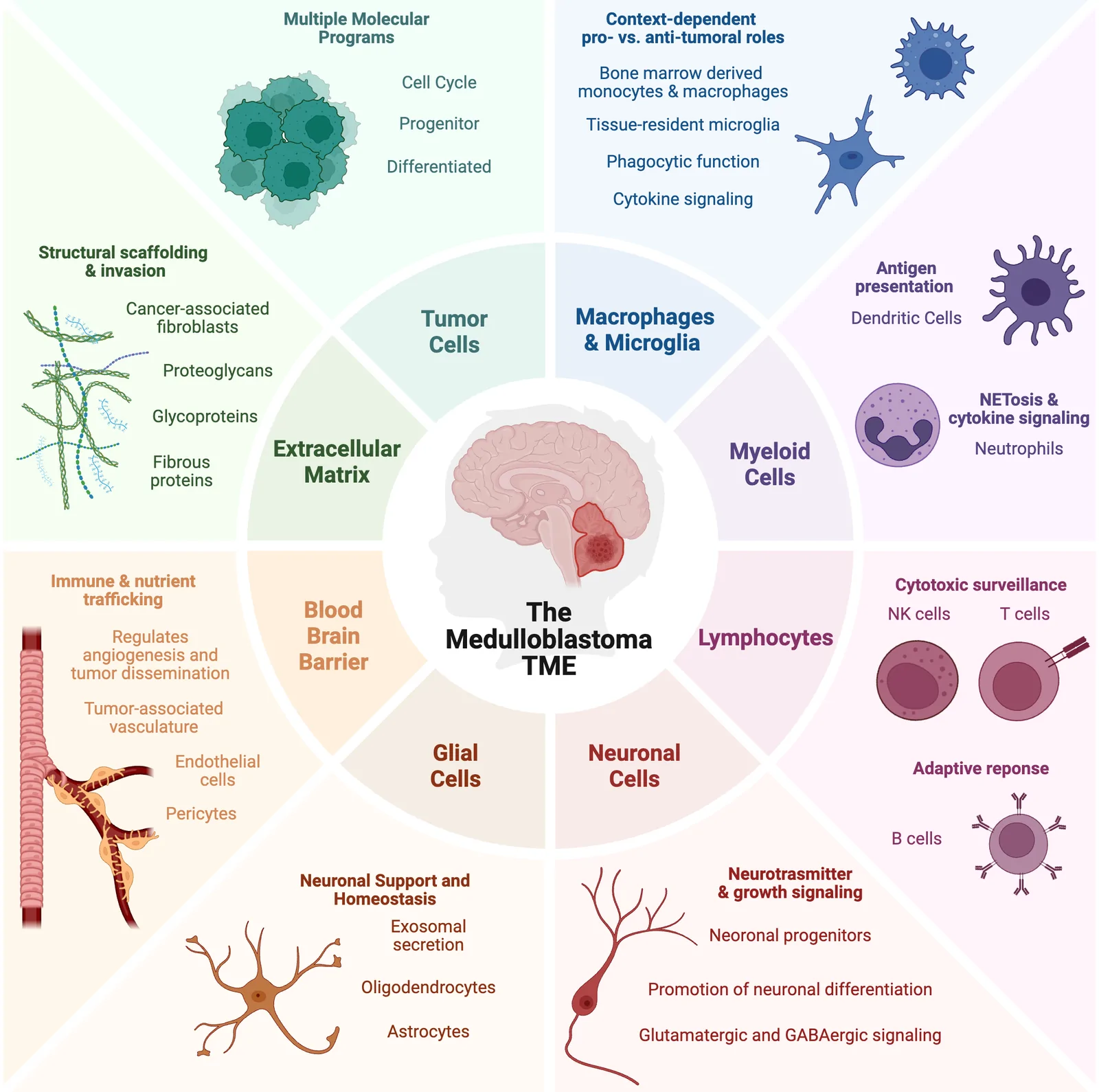
The composition of human medulloblastoma tumor microenvironment within the cerebellum, which collectively constitutes tumor cell extrinsic factors modulating disease progression, proliferation, metastasis, and response to chemotherapy. Along with the constituent cell types, extracellular matrix scaffolding, neuronal signaling, and molecular paracrine signaling compounds all contribute to form the complex contextual environment in which tumors arise and are treated.

The MB tumor microenvironment (MB-TME) is a complex and understudied source of disease heterogeneity, characterized by blood vessels and endothelium, extracellular matrix (ECM), astrocytes, oligodendrocytes, differing populations of neurons, as well as cytokines and tumor-infiltrating immune cells comprising of microglia/macrophage and lymphocyte populations (**Figure 1**) (8). How the TME modulates disease biology, whether by supporting cancer cell survival or through its anti-neoplastic effects remains poorly understood in MB. To date, therapeutic strategies in MB have yet to leverage vulnerabilities in TME crosstalk. In this review, we examine the emerging understandings of the MB TME and discuss early investigational strategies aimed at targeting the TME to improve outcomes.

## 2 Tumor microenvironment of medulloblastoma

### 2.1 Tissue architecture

The overall MB tissue architecture is described through histopathological classification in the current 2021 WHO CNS tumors update, and includes: (1) classic, defined by a characteristic densely-packed and poorly-differentiated small round blue cell tumor with occasional patterns of differentiated tumor cells surrounding a central region of neuropil termed Homer Wright Rosettes, (2) desmoplastic nodular, characterized by nodular, reticulin-free zones surrounded by collagenous matrix containing highly proliferative tumor, (3) MB with extensive nodularity (MBEN), with increased neuropil matrix compared to desmoplastic nodular and only scant internodular tissue, and (4) large cell/anaplastic, exhibiting large and pleomorphic nuclei with concomitant high mitotic and apoptotic rates (9).

**Figure 2:**
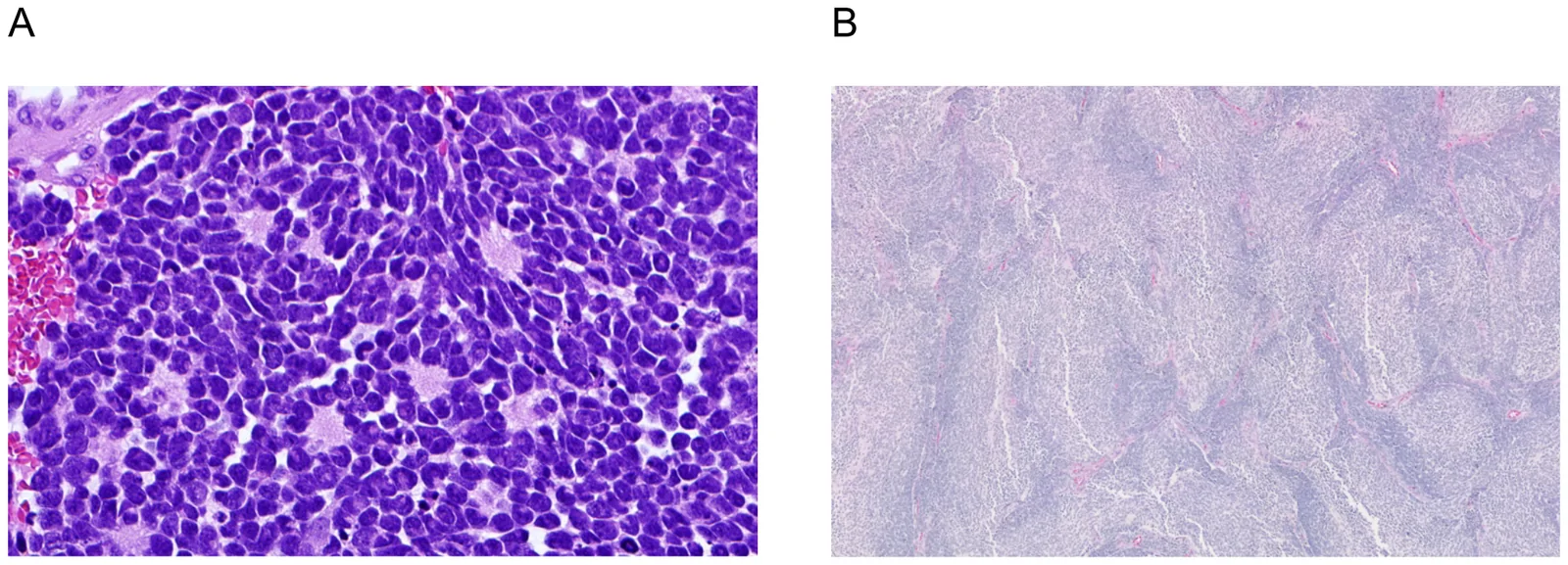
Representative H&Es of medulloblastoma tumor microenvironment (MB-TME) illustrating the spatial dependence of cancer cell biology. Images obtained from patients treated at Children’s Healthcare of Atlanta, Atlanta, GA. **(A)** shows Homer-Wright Rosettes, with multiple MB cells arranged circumferentially around a central clearing of neuropil. **(B)** shows desmoplastic/nodular histology which is defined by nodular areas of increased differentiated cells with internodular zones of high proliferation.

Within these histological subgroups, spatial differences are readily observed under light microscopy. Classic histologic subtype MB may contain nodules of neurocytic differentiation. Both desmoplastic/nodular and MBEN subtypes show nodular zones surrounded by more densely packed and highly proliferative internodular zones (**Figure 2**). Histologically informed microdissection-based sequencing in MBEN corroborates the spatial heterogeneity observed in light microscopy on the molecular scale, with nodular niches containing neuronally differentiated malignant cells compared to internodular zones characterized by stroma and proliferating cerebellar granule precursor malignant cells (10).

Collectively, the current literature recognizes spatial heterogeneity on the overall TME architecture both in histological appearance and molecular composition. Therefore, studies on the MB-TME should ideally be interpreted within the context of the histological source. On either the histological or molecular scale, this heterogeneity has yet to be meaningfully translated to therapy.

### 2.2 Cellular and molecular composition

Cellular composition of the MB-TME is informed by several sources, including appearance on light microscopy together with marker-based and sequencing-based approaches. Histologic section readily identifies tumor, immune, and glial cells along with interspersed vascularity. Macrophage and microglial immunohistochemical markers have long been recognized in MB (11, 12). Similarly, a role for astrocytes in malignant TME biology can be inferred from the observation that injury from CNS cancers incur a reactive gliosis (13).

Single-cell RNA sequencing (scRNA-seq) technology has been transformative in determining TME cell composition by enabling an unbiased, whole-transcriptomic approach to cellular characterization. Recently, a growing body of scRNA-seq research has given new insight into the MB-TME. The first comprehensive scRNA-seq reference in human MB disease spanning all molecular subtypes was published in 2019 (14). The study, intended to focus on malignant cell populations, was broad in the inclusion of samples both at diagnosis and at recurrence. Among the insights reported include evidence supporting unipolar brush cells as a putative origin for G4 MB. However, the systematic exclusion of non-malignant cells leaves the gene expression profile of these populations unexplored.

This gap in knowledge was mitigated by a 2022 study which expanded the MB scRNA-seq literature with increasing number of samples, mouse models for cross-species comparison, and the inclusion of non-malignant immune populations (15). Immune cells in the TME demonstrated a majority myeloid population followed by a smaller lymphoid population. Notably, myeloid cells were unique in that its proportion of the TME composition markedly varied by molecular subgroup with increased abundance in SHH MB. A diversity in gene expression profile was recognized within myeloid cells, however, whether these cells belonged to tissue-resident microglia or bone-marrow derived circulating populations was difficult to distinguish due to overlapping of gene expression markers. The finding of gene expression overlap corroborates other studies of CNS microglia and macrophage (16), underscoring a limitation of transcriptomic-based methods in studying subpopulations of myeloid cells within the MB-TME.

Overall, biological insight derived from scRNA-seq based technologies should be interpreted carefully and understood considering the innate limitations of the platform. First, artifacts may be introduced during library preparation and tissue handling. Cell isolation and dissociation required for scRNA-seq disrupts cells from their native cellular environment, and enzymatic treatment may induce artifactual changes in gene expression such as in stress-response related genes or neuronal activation (17). Second, scRNA-seq dataset feature significant degrees of sparsity, or zero measurements, across cells and genes (18). Consequently, it may be difficult to distinguish true absence of transcript from technical limitations to sensitivity. Additional scRNA-seq datasets, if corroborating the results, would allow more confidence in the robustness and generalizability of findings.

TME molecular composition is ideally understood through a multi-dimensional approach. This is supported by several studies across cancer types that highlight the variability in concordance between mRNA and protein-level techniques (19, 20). Compared to genomic and transcriptomic profiling, proteomic studies are a relatively newer field. In MB, early proteomic data corroborate the division into the four molecularly defined subgroups, and further show differential protein enrichment across these subgroups (21). High differential upregulation of PMEL (Melanocyte protein) and FBN2 (Fibrillin2) was demonstrated in WNT MB, in contrast to SYNGR2 (Synaptogryin-2) in SHH MB and GFAP in non-WNT/SHH disease. Synaptogyrins are a family of tyrosine-phosphorylated integral membrane proteins notably involved in synaptic vesicles at nerve terminals (22), but otherwise how these proteins influence the MB-TME remains incompletely understood. In addition to individual proteins, collective pathways also show differential enrichment, including angiogenesis in WNT, cell-cycle in SHH, and apoptosis signaling pathway for non-WNT/SHH. Across subtypes two broad molecular patterns are described, a transcriptional/replicative pattern and a synaptic/immunological pattern, with the latter hypothesized to be more dependent on external modulation from the MB-TME (23).

### 2.3 Spatial molecular profile

A limitation of scRNA-seq is that spatial relationship data are lost. At present, spatially informed transcriptomics in MB-TME remains a nascent field with limited studies. One study, conducted in an East Asian population, recapitulated the biological programs first defined by scRNA-seq but also highlighted the heterogeneity in spatial architecture and tissue organization (24). However, at present there remain challenges to spatial resolution with the current spatial-sequencing platforms. Spatial spot sizes, or “voxels”, may contain more than a single cell, therefore providing aggregate gene expression of a cell mixture. The added spatial dimension to the dataset also exacerbates concerns with respect to sparsity. These concerns are best addressed by additional sequencing datasets that may challenge or corroborate existing ones, and validation through studies orthogonal to the transcriptome.

On a protein and lipid scale, three-dimensional matrix-assisted laser desorption/ionization mass spectrometry imaging (MALDI-MSI) studies on MB-bearing transgenic mice show a spatially-heterogenous lipid profile with specific lipids corresponding to metastasizing boundaries (25). However, whether this finding translates to human disease is unknown, as similar studies have yet to be completed in human MB.

In aggregate, early studies agree on both the intra- and inter-tumoral spatial heterogeneity within the MB-TME, but biological insight overall remains limited by the paucity of studies.

## 3 Tumor microenvironment role in medulloblastoma tumorigenesis

### 3.1 Oncogenesis and tumor formation

Once considered a unitary disease, MB is now recognized to have distinct pathogenesis and cell of origins depending on molecular subtype. For example, SHH MB is currently thought to arise from the granule neuron progenitor cells in the upper rhombic lip, which migrate outward to form the external granular layer of the developing cerebellum (26). Mutations within the SHH pathway of these cells may then trigger tumorigenesis. The WNT-activated MB subgroup, in contrast, is characterized by aberrant activation of the effector protein CTNNB1 in approximately 85-90% of cases. Evidence for their cell of origin derives primarily from their gene expression and methylation profile, showing resemblance to progenitor cells from the caudal rhombic lip and dorsal brainstem-derived neuron lineage.

Relatively less is known about the oncogenesis of the non-WNT/SHH subgroups G3 and G4, in part due to the increased difficulty in distinctly isolating these subgroups as a result of their significant overlap in gene expression. No distinct mutation or single aberrant genetic alterations characterize G3 and G4 tumors. Rather, recent studies show G3 and G4 disease existing along an expression continuum rather than as wholly distinct disease entities, with this continuum reflected in early human cerebellar development from earlier embryologic stages mirroring more archetypical G3 to later stages more similar to G4 (27). Cross-species scRNA-seq data suggests unipolar brush cells are the cell of origin for G3 and G4 MB (14).

### 3.2 Oncogenic signaling and modulation of the TME

There are likely several mechanisms by which oncogenic signaling modulates the MB-TME and in turn influences tumor initiation and growth. For example, in murine models SHH MB, cancer cells release Shh ligands that act not only in an autocrine loop, but also in a paracrine manner to regulate the function of the surrounding stromal and immune cells of the TME. Specifically, Shh paracrine signaling induces stromal cell proliferation resulting in desmoplasia and alters the deposition of ECM components like collagen and hyaluronan to create an environment that supports tumor cell adhesion, survival and colony formation (28). Paracrine signaling by Shh can also mediate a metabolic switch toward lipid production that favors MB cells proliferation and regulate stromal cell secretion of placental growth factor (PIGF) to stimulate new blood vessel growth that is essential for continued tumor growth (29). Shh signaling has also been shown to remodel the immune microenvironment, providing a supportive niche for immune evasion (28). Shh ligands were also found to attract immunosuppressive M2-polarized tumor associated macrophages (TAMs) to the tumor, which inhibit the recruitment and activation of anti-tumor CD8+ cytotoxic T cells by suppressing chemokines like CXCL9/10 and promoting PD-L1 expression on cancer cells (30, 31). Shh activity also increases Tregs and other anti-inflammatory mediators such as IL-10 and TGF-β that further contribute to the immunosuppressive TME (32).

In murine models of WNT MB, WNT signaling induces M2-polarized TAMs resulting in immune tolerance via the suppression of cytotoxic T cell infiltration and stimulates angiogenesis (33). WNT signaling further creates a tumor-promoting environment by recruiting myeloid derived suppressor cells to the tumor (34) and engages in complex crosstalk with pro-survival pathways such as TGF-β, Hippo, and Notch, to enable cancer progression (35). Animal models have shown mechanistically that WNT signaling in the cerebellum is regulated by the activation of Norrin/Frizzled4 (Fzd4) within the endothelial cells of the leptomeninges (36), and based on scRNA-seq profiling, Fzd4 functions to maintain the activation of meningeal macrophages that support tumor survival (37).

In human G3 and G4 MB, the oncogenes *MYC* and *MYCN* are commonly amplified (38). Amplification of these oncogenes in animal models of these tumors results in the release of extracellular vesicles that promote the expression of immune suppressive molecules, including PD-L1 and CD47, which inhibit cytotoxic T cell responses and promote the recruitment of TAMs to the local environment (39). *MYC* is also a key driver of angiogenesis by stimulating the production of vascular endothelial growth factor (VEGF) and can act to remodel the surrounding stromal cells to further support tumor growth, invasion, and metastasis (40).

*TP53*, a tumor suppressor gene often designated as the “guardian of the genome”, is similar to MYC in its widespread impact on cancer cell biology. Germline and somatic mutations of *TP53* are particularly associated with SHH MB. The ubiquitin ligase MDM2 mediates the negative regulation of the p53 tumor suppression protein (41). Patient derived xenograft (PDX) models show inhibition of MDM2 reactivates p53 protein function in SHH-MB and has been promoted as a novel target of therapy (42). All of these complex interactions observed in murine models of WNT, SHH, G3, and G4 MB require human validation before translation to therapeutic intervention can be considered. To address this issue, combined multi-omics studies analyzing the tumor tissue, CSF, and blood in patients with MB are underway.

### 3.3 Role of astrocytes and neurotransmitters

**Figure 3:**
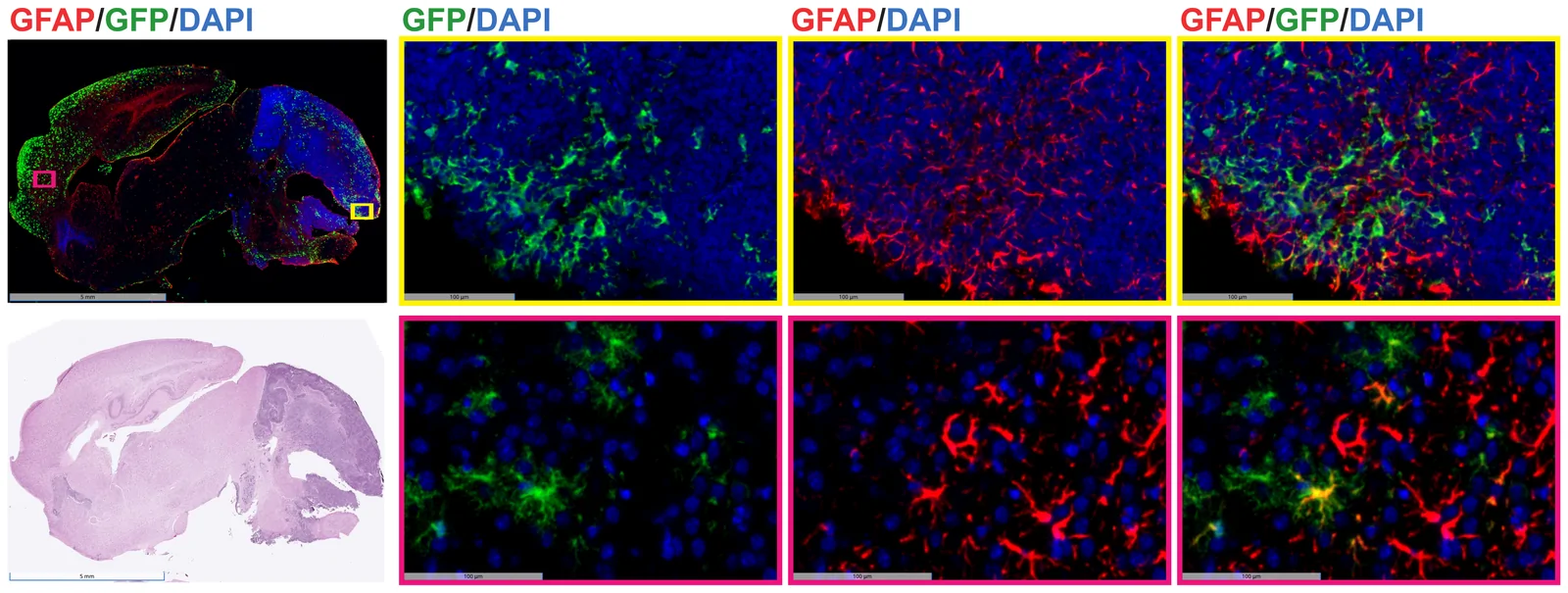
Original data of immunostaining of adeno-associated virus (AAV) construct in GFAP-CRE/SmoM2 medulloblastoma mouse model showing astrocytes with GFP (green), GFAP (red) and DAPI (blue), representative regions of tumor (upper row) and normal murine brain (lower row) shown.

Under physiologic conditions, astrocytes, marked by GFAP and S100B expression, are widely distributed throughout the brain and the most abundant glial population. Astrocytes play a key role in supporting the proliferation and migration of cerebellar granule neuron precursors. Developmentally, cerebellar astrocytes support the proliferation and migration of granule cell precursors, the proposed cell of origin in SHH MB (43). GFAP+ tumor associated astrocytes (TAAs) can be demonstrated in murine models of MB through immunostaining (**Figure 3**).

The role of TAAs in the MB-TME is an emerging area of study. Current evidence supports TAAs as a distinct population from astrocytes in healthy brain by their increased activation and expression of GFAP, along with other paracrine signaling within the TME that may promote MB growth (44, 45). Within malignant cells, subpopulations of MB cells show transcriptional similarity to astrocytes (10), suggesting a reciprocal crosstalk and influence between the two populations.

In murine models of MB, TAAs also secrete Shh ligands that induce synthesis of inflammatory leukotrienes, which leads to the expression of Nestin, a cytoskeletal protein critical for SHH MB growth (44, 46), and lipocalin-2 that in turn activates the oncogenic STAT3 signaling pathway via insulin-like growth factor binding protein-2 (IGFBP2) (47, 48). While these functional interactions have been resolved in murine models of SHH MB, it remains to be confirmed whether this crosstalk similarly occurs in human MB and across molecular subgroups or is restricted to SHH MB. Validation of these interactions will require next generation scRNA-seq and spatial sequencing across large sample cohorts of molecularly diverse human MB and in more humanoid models in which the functional mechanisms can be studied.

The role of neurotransmission in the MB-TME is increasingly recognized as well, particularly that of GABAergic inhibitory interneurons. GABA the most common inhibitory neurotransmitter in the CNS, is predicted to play an active role in regulating the development and function of cerebellar neurons (49). Cerebellar granule cells express GABA receptors, and receptor activity in part orchestrates differentiation. It is also worth noting that human G3 MB, the most aggressive molecular subtype, show elevated levels of GABRA5 expression (50). The role of neurotransmission in MB pathogenesis is supported through murine GABA metabolism studies that show GABA metabolism was decreased globally in the MB-TME, and even more so for more aggressive subtypes (51).

### 3.4 Metabolic dysregulation

Aberrant cellular metabolism promoting malignant proliferation is a hallmark of cancer biology and may be modulated by the TME. Metabolic alterations have specifically been recognized in human MB including changes in major pathways such as glycolysis and fatty acid synthesis (52, 53).

The Warburg effect, common across many cancers, describes a metabolic dysregulation whereby malignant cells demonstrate a preference for glycolysis and lactic acid fermentation rather than oxidative phosphorylation even in the presence of oxygen (54, 55). The relative hypoxic conditions within MB-TME promotes hypoxia-inducible factor 1a (HIF-1a), which further reinforces glycolysis and suppresses mitochondrial metabolism (56). Several trials have sought to exploit this difference, but thus far have yet to yield efficacious therapy.

**Figure 4:**
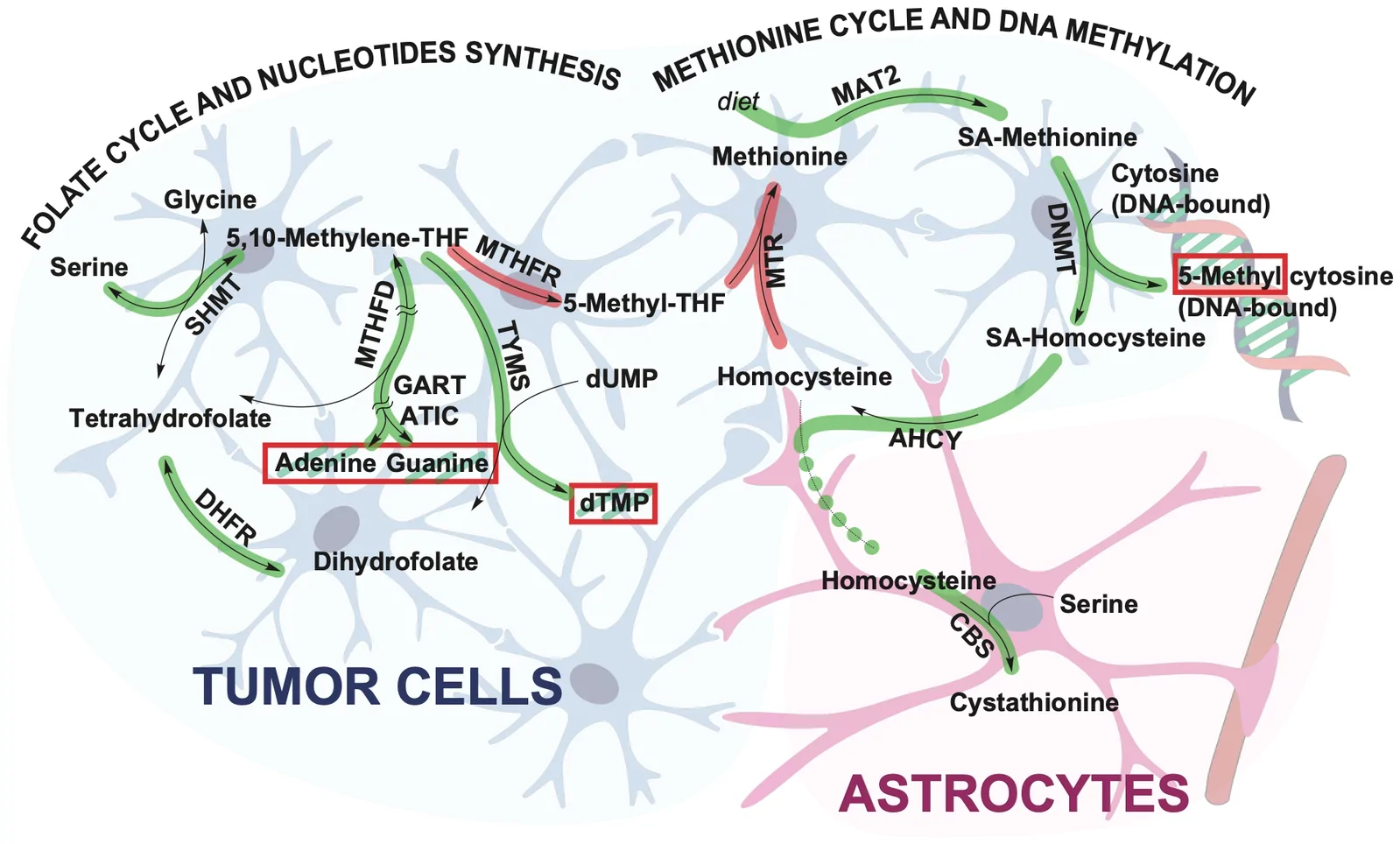
Malignant MB cells interact with astrocytes in the MB-TME and show dysregulated folate cycle and methionine cycle metabolism to promote proliferation. Upregulated pathways in malignancies are shown in green and red shows slowed pathways. Purine and pyrimidine synthesis rely on the folate cycle powered by one-carbon metabolism and compete with the methyl cycle for the methylated folates. Tumor cells prioritize nucleotide synthesis. In accommodating the increased demands for DNA methylation and to decouple from the folate cycle, tumor cells rely on the dietary methionine and run methionine cycle in a linear fashion resulting with toxic byproduct – homocysteine. Instead of re-methylation back to methionine, tumor cells excrete it into the MB-TME where it is taken up by astrocytes converted to the harmless cystathionine. In decoupling the two cycles, tumor cells are able to run both pathways at higher capacity promoting malignant growth.

Cancer cells use two high-priority pathways that are in competition for one-carbon methyl groups. These pathways include nucleotide synthesis for DNA replication and DNA methylation (**Figure 4**). Malignancies rewire metabolism and show different methylation profiles, with high demand for nucleotide synthesis. DNA synthesis and methylation are decoupled such that cancer cells can maximize both processes as opposed to non-malignant cells where synthesis and methylation pathways are more closely regulated and intertwined.

Methotrexate disrupts folate cycle and halts nucleotide synthesis in actively proliferating cells. Recently, the Children’s Oncology Group trial ACNS0334 demonstrated a survival benefit for a subset of MB patients who had received folate inhibition through administration of methotrexate (57). Further, nucleotide synthesis relies on carbon supply using two major enzymes: SHMT1/2 and MDHFD 1/2 (58). Inhibitors of these enzymes have been tested for various cancers including brain cancers, but these inhibitors have yet to be studied in MB (59, 60).

Overall, differential metabolism between tumor and non-tumor TME remains poorly understood. Targeted molecular inhibition leveraging these metabolic differences have been studied as a potential therapeutic strategy. However, the ubiquity of pathways in non-malignant cells and the finding that cancer cells can often adapt through activation of bypassing pathways poses major challenges to this approach.

### 3.5 Immune suppression, role of tumor associated macrophage and microglia

Early MB sequencing studies revealed a relative lack of immune cell infiltration within the MB-TME, leading to the overall characterization of MB as an immunologically cold tumor (61). However, even the few immune infiltrating cells show subgroup specific characteristics (62). WNT MB was found to be enriched for supportive stromal cells such as pericytes, astrocytes, and oligodendrocytes, and demonstrated increased features of angiogenesis. In contrast, SHH, G3, and G4 subtypes show immunosuppressive TMEs dominated by TAMs and exhausted T cells. Both sequencing and non-sequencing-based approaches converge on TAMs and microglia being proportionally the most abundant tumor-infiltrating immune cells within the MB-TME (15, 63, 64). SHH tumors demonstrate a proportionally higher infiltration of CD163+ TAMs compared to G3 and G4 (65).

Myeloid-specific subtyping of human MB revealed 11 functionally distinct TAM clusters encompassing WNT-enriched microglia-derived M1-polarized subsets with anti-tumor activity, SHH-specific M2-like classical TAMs promoting immune evasion, and G4-associated ECM-remodeling TAMs driving malignancy (62). In non-WNT tumor subtypes, T cell profiling also identified both exhausted CD8+ cell populations and enrichment for CD4+ regulatory T cells, indicative of an immunosuppressive TME. Trajectory analysis suggests myeloid differentiation routes starting from proliferative TAMs to terminal immunosuppressive subsets, and uncovers subtype-specific regulatory transcription factors, such as XBP1 and STAT6. Intercellular communication inference through co-expression of known ligand-receptor pairs identifies MIF-CD74 and MDK-LRP1 as potentially key mediators of tumor-TME crosstalk in non-WNT subtypes.

Historically, TAMs have been distinguished by pro-inflammatory M1 versus anti-inflammatory M2 polarity, although the prognostic significance of macrophage polarity in MB remains an unresolved frontier. In murine models of SHH MB the polarization of microglia to TAMs is regulated by interleukin-4 (IL-4) secreted by TAAs, and these TAMs in turn appear to promote tumor progression through secretion of insulin-like growth factor 1 (IGF-1) (63). Recent studies in adult carcinoma implicate the CXCL9:SPP1 macrophage polarity as a prognostic indicator with elevated CXCL9 to SPP1 ratio predicting favorable survival (66). Corroborating this is the finding that SPP1+ macrophage promotes worse clinical outcomes in lung cancer (67, 68). SPP1 positivity has also been shown in glial tumors (69). Whether this macrophage polarity is of prognostic or therapeutic consequence in human MB warrants further study.

Tumor-infiltrating lymphocytes (TILs) are a rarer infiltrating immune population. The main TIL subsets in human MB are CD3+ and CD8+ T-cells, however, the degree of TIL infiltration was not shown to influence survival outcomes (70). Another study showed a significant enrichment of T- and B-cells in the SHH subtype of MB compared to the WNT, G3 and G4 tumors (63). In contrast, infiltrating B-cells and neutrophils were shown to be more abundant in G4 tumors, while WNT MB were found to have relatively increased levels of NK cells (71). The role these cell populations play in MB pathogenesis and response to treatment remains an area of active investigation.

## 4 Tumor microenvironment in progression and treatment resistance

### 4.1 Mechanisms of TME-mediated metastasis

Leptomeningeal metastatic disease (LMD) is the leading cause of morbidity and mortality in patients across all MB subtypes. A number of animal model studies identify cell signaling interactions between tumor cells and their TME that may drive MB metastasis. The mechanisms reported for driving metastasis, however, have been elucidated almost entirely using preclinical murine disease models and have yet to be validated in human MB. To address this concern, various ongoing studies use serial sampling of MB patient biofluids to confirm and further explore the key interactions that have been described.

Extracellular vesicles (ECVs) shed by tumor cells into the MB-TME may be one mechanism by which tumors metastasize. In preclinical models of MB, ECVs facilitate ECM degradation and remodeling within the MB-TME through carrying enzymes like matrix metalloproteinase-2 (MMP-2), and in doing so may create a milieu conducive for promoting cellular migration and stromal invasion (72). Supporting this hypothesis is the correlation of increased ECV and MMP-2 secretion with more aggressive tumors. Metastatic MB cells secrete PDGF into the TME, which acts to locally recruit MB-MAFs. MB-MAFs in turn secrete bone morphogenic proteins which together with MB-MAFs may nurture the implantation and colony formation of the migrating MB cells to the leptomeninges.

Preclinical murine models also suggests that once MB cells have migrated to the leptomeninges, the establishment of LMD is closely associated with neural paracrine regulation of GABA secretion (51). GABA catabolism is regulated by the enzyme GABA transaminase (ABAT), which allows neurons and astrocytes to utilize GABA as an alternative energy source during cerebellar development. In the pathogenesis of MB LMD, metabolic changes within the TME may regulate ABAT expression whereby nutrient-poor conditions result in the upregulation of ABAT expression. Invading MB cells require ABAT to maintain their viability in the cerebrospinal fluid using GABA as an energy source. However, it remains to be seen whether this mechanism of LMD in murine models accurately reflects the process in humans before therapeutic interventions disrupting these pathways could be considered.

The blood-brain-barrier (BBB) within the MB-TME, by regulating both access to critical nutrients and exposure to anti neoplastic agents, may also influence the development of LMD. BBB integrity varies by MB subgroup (73). Strikingly, it was demonstrated in animal models of MB that local inflammation mediated by therapeutic irradiation results in the recruitment of immune cells to the apoptotic tumor cells, which in turn opens up the BBB allowing for the intravasation of viable MB cells to facilitate LMD (74).

Finally, a preclinical murine model demonstrates a hematogenous route for MB metastasis and proposes (CTCs) that have detached from the primary tumor and extravasated into the systemic circulation as a mechanism (75). In this study, MB cells injected into one mouse connected to another mouse by a parabiotic vascular anastomosis were found to circulate and hone to the CNS of the recipient mouse to form LMD. Notably, the chemokine CCL2 was found differentially expressed in LMD compared to primary tumor on microarray. Subsequent murine experiments showed expression of CCL2 led to increased incidence of spinal LMD in cell line xenografts that otherwise are poorly metastatic. A follow-up study of the LMD TME show interaction of metastatic tumor cells with meningeal fibroblasts, which in turn promotes tumor colonization of the leptomeninges (73). The characteristics of MB CTCs on the molecular level allowing for survival in circulation remain unclear and are a focus of ongoing investigations. Similarly, how CTCs interact with the TME or other circulating cell types is poorly understood.

### 4.2 TME contribution to therapeutic resistance

Despite management with aggressive multimodal therapy, relapse and progressive disease remain a significant concern in MB, underscoring a need to understand and overcome treatment resistance. While considered leakier at the tumor interface, the selectively permeable BBB remains a major barrier to drug delivery both clinically and in pre-clinical investigation. Similarly to the BBB, evidence supports cancer associated fibroblast in TME creates stiff ECM that may impair immune cell movement, and thus contribute to the relative immunosuppressed TME (76). Application of sequential pulses of high- and low-voltage electric fields is currently studied in mice as a means of reducing ECM density and stiffness, thus exposing tumor to cytotoxic anti-tumor immune cells (77). Future spatially informed studies of the MB-TME can assist in identifying how readily novel agents can permeate the TME with respect to the BBB and other stromal components.

Chemotherapy typically relies on cell cycling. Quiescence with respect to cell cycle has been shown to be one mechanism of therapy resistance. The finding of spatial heterogeneity by transcriptomic program across cancer cells in the MB-TME suggests that quiescent spatial niches may contribute to poor treatment outcomes. This is supported by the finding that brain cancer stem cells, a subpopulation predicted to show increased innate resistance to treatment, spatially co-localizes with endothelium (78). Collectively, the findings suggest the existence of subpopulations of therapy-resistant MB cells within specific spatial niches. Further studies are needed to verify this hypothesis, and if proven correct, may be exploited as a novel therapeutic strategy.

Comparative gene expression analysis from *in vitro* and PDX MB models have shown distinct upregulation of Myc targets, oxidative phosphorylation, nitric oxide synthase, and superoxide metabolism after relapse (79). The study further implicates a novel protein BPIFB4 that was found to be upregulated upon relapse. Targeting the downstream substrate endothelial nitric oxide synthase inhibited growth of human G3 MB cell lines. Despite these intriguing findings, our understanding of the precise role and impact of the MB-TME on treatment resistance remain limited to a few studies and mainly in murine preclinical models of MB.

TME cytokines may also play a role in the proliferation of tumor and therapy resistance. In glioma, data from multi-national transcriptomic gene expression atlases showed that a long non-coding RNA (lncRNA) signature relating the transforming growth factor-β (TGF-β) correlated with survival (80). Targeting TME cytokines is explored This result has yet to be verified in MB. If similar prognostically relevant cytokine signatures are discovered, the finding underscores the need for further studies understanding the role of the immune TME in MB. Enduring response.

Understanding how the human MB-TME evolves over the course of treatment and upon relapse would provide a critical piece of knowledge to the study of TME with respect to therapy resistance. However, a key barrier to this is the limited availability of surgical samples following initial diagnostic tumor resection, as there is rarely a clinical indication to obtain such samples. Further, it may be difficult to distinguish innate tumor cell progression from therapy induced changes. However, it’s reasonable to predict that selective pressure from antineoplastic therapy may promote evolution towards therapy resistance, whether by increasing proliferation, expressing treatment ejecting pumps, or by bypassing the mechanism of action.

## 5 Therapeutic implication of targeting the TME in medulloblastoma

### 5.1 Immune and cellular therapies

Therapeutic strategies exploiting differences in TME biology is a promising direction for novel therapy in MB. A variety of trials targeting cell surface proteins of either immune or tumor cells using monoclonal antibodies or Chimeric Antigen Receptor T-Cells (CAR-T) are under investigation. Other strategies for novel therapy may include modulation of intracellular pathways or altering the immune microenvironment.

Activated T cells upregulate immune checkpoint molecules, such as PD1 and CTLA4, which in turn prevent autoimmune destruction. However, many cancers upregulate PD-L1 to exploit this checkpoint signaling as a means of immune evasion. Immune checkpoint inhibition therapies are designed to block this process. Nivolumab (anti-PD1) and ipilumumab (anti-CTLA4), are being investigated for the treatment of CNS tumors both in adults and pediatric patients. Nivolumab and ipilimumab showed intracranial activity in adults with metastatic melanoma, supporting their potential as CNS immunotherapy (81). However, a pediatric trial of nivolumab with ipilimumab failed to show clinical benefit in pediatric patients with newly diagnosed diffuse intrinsic pontine glioma (DIPG) or relapsed/refractory high-grade brain tumors (82). Results of the efficacy of these two agents have yet to be reported in MB, although clinical trial studies are being conducted.

In a separate pediatric phase I/II trial, INFORM2, checkpoint inhibition with nivolumab is being investigated in combination with the histone deacetylase (HDAC) inhibitor entinostat for patients ages 6–21 years with high-risk relapsed or refractory solid and CNS malignancies including those with ATRT-MYC subgroup or MYC/MYCN amplified MB (NCT03838042). At present, results have yet to be published after peer review. HDAC inhibition has been shown to modify T cell regulation through reduction in myeloid-derived suppressor cells, enhancing tumor-infiltrating T cell function. Using the same strategy in adults, nivolumab and entinostat are being investigated in a phase II trial for patients with recurrent or progressive rare CNS tumors (NCT03173950). Lastly, pembrolizumab, a PD1 inhibitor, is currently under investigation in a pediatric phase I trial for recurrent or refractory MB, as well as recurrent, progressive, or refractory high-grade gliomas, hypermutated brain tumors, and ependymomas (NCT02359565).

Another study aimed at interrupting immune tolerance is the investigational agent indoximod. Indoximod targets the indoleamine 2,3-dioxygenase (IDO) pathway. Preclinically, the IDO pathway has been shown to contribute to tolerance of antigens derived from apoptotic cells, a potential mechanism of immune-escape (83). In a first-in-child phase I trial, indoximod was found to be well tolerated without a maximum tolerated dose reached with 4 patients who remained free of disease over three years off-therapy (84). A subsequent phase II trial (NCT04049669), incorporating the addition of temozolomide for patients ages 3–21 years with either recurrent brain cancer including MB or newly diagnosed DIPG, has been completed but to date a final report has not yet been published. The interim analysis replicated the phase 1 results with as estimated OS of 11.5 months for recurrent MB (84).

Outside of inhibition of immunosuppression pathways, stimulation of anti-tumor immune cells, is also under investigation. Sotigalimab (APX005M), a fully-humanized monoclonal antibody, is a CD40 agonist that helps to stimulate proliferation of tumor-directed T cells (85). In addition, sotigalimab mediates a direct cytotoxic effect on CD40+ tumor cells without damaging CD40+ immune cells. The combination of sotigalimab and PD-1 inhibition was found to yield durable response in anti-PD-1 resistant melanoma (86). Future trials are needed to evaluate this strategy in MB.

Additional tumor-directed strategies include targeting proteins upregulated on MB tumor cells, including B7-H3. As a member of the B7 co-stimulatory family, B7-H3 is important for regulating the proliferation and function of immune cells (87). CD4+CD25+ Tregs have been shown to upregulate B7-H3 within the TME, inducing an immune-tolerant response (88). Preclinical studies show B7-H3 is not only found on MB tumor cells but in other components of the MB-TME. Thus, in clinical studies, B7-H3 is being targeted with hopes of both eliminating the immunosuppressive response and directly damaging the tumor cells.

B7-H3 has shown early signal as a target not just in pre-clinical studies, but in early phase clinical trials for high-grade glioma, including DIPG. The Phase I clinical trial BrainChild-03 (NCT04185038) studied CAR-T cells targeting B7-H3 (33, 34), demonstrating safety when delivered intraventricularly to patients with high-grade gliomas (89, 90). It also demonstrated early signals of efficacy in patients with DIPG, leading to the design of a Phase II trial of loco-regional delivery of B7-H3 CAR T-cells to patients with DIPG. In addition to BrainChild-03 targeting B7-H3, BrainChild-04 (NCT05768880) expanded upon prior trials, serving as the first multi-target CAR-T trial in MB, infusing a single population of CAR T-cells that simultaneously recognizes four different antigens: B7-H3, EGFR806, HER2, and IL13-zetakine. B7-H3 is also being targeted with a monoclonal antibody, 131I-Omburtamab through a phase 2 trial of pediatric patients with relapsed MB or ependymoma (NCT04743661). Additional CAR-T trials are underway, targeting cell surface antigen upregulated on MB and high-grade brain tumors, including GPC2 (trials NCT0708002), GD2 (NCT05298995), and IL13Ra2 (NCT04661384).

Historically, multimodal chemotherapy with diverse mechanisms of action yielded more favorable results compared to single-agent chemotherapy. It may be inferred that a similar principal applies with novel immune or cellular therapies. In a pediatric phase I trial, ibrutinib, a Bruton’s tyrosine kinase inhibitor, is being studied in combination with indoximod and oral chemotherapy with the intention of improving clinical response while managing overlapping toxicities (NCT05106296). Further, literature in hematologic malignancy predicts that maintaining immune memory through T memory cells is key for durable disease control (91). Future studies of the MB-TME should similarly study the role of T memory cells and how it changes throughout immunotherapy.

### 5.2 Targeting of neurotransmitter signaling and protein-protein interactions

Innervation and neurotransmitter signaling influences malignant biology and therefore may represent an opportunity for therapeutic intervention, even outside of the CNS (92). One preclinical study showed simulation of the GABA_B_ receptor in lung and colon resulted in increased β-catenin signaling and subsequently to tumor growth and suppression of CD8+ T-cell tumor infiltration.

Specific to MB, transcriptome-level evidence in human MB suggests that higher expression of GABA_A_ receptor β subunit genes (*GABRB1*, *GABRB2*, and *GABRB3*) correlated with longer survival (93). Conversely, high expression of *GABRA5* gene, encoding the α-subunit of the GABAA receptor complex, was seen in aggressive MYC-driven MB, with cell line data showing that targeting GABAA receptor resulted in decreased tumor cell survival (94). This pathway may thus serve as an opportunity for future clinical investigation.

As previously noted, astrocyte-derived Shh ligand promotes MB proliferation in preclinical murine models of the disease. Notably, depletion of astrocytes suppressed MB growth (44), underscoring their therapeutic potential. Beyond Shh signaling, TAAs can also promote tumor progression and metastasis through cytokine signaling. Astrocyte-derived CCL2 was implicated in maintaining stem-like properties and pro-metastatic behavior, motivating investigation of CCR2-CCL2 signaling as a targetable interaction within the leptomeningeal niche (95). Another potentially targetable TME interaction is the G-protein coupled receptor CXCR4, which interacts with the tumor-promoting cytokine CXCL12, primarily expressed by the endothelium of tumor-associated blood vessels (96). Pharmacologic disruption of CXCL12-CXCR4 reduced MB progression in preclinical PDX models.

Protein-protein inhibition through small peptides demonstrate high affinity and specificity, making them a promising direction for new drug discovery. However, they are often highly susceptible to proteolytic degradation. The development of mirror-image proteins may potentially overcome this limitation (97). Mirror-image proteins, which can only be chemically synthesized, leverages the finding that all biological molecules share the same chemical chirality or ‘handedness’. Thus, the mirror image may retain the on target drug properties while being resistant to proteolytic cleavage.

Collectively, these studies highlight protein-protein interactions in the MB-TME that may be explored as the target of novel therapies. Due to the complexity and evidence of heterogeneity in the MB-TME across subgroups, successful translation of these findings will benefit from further subgroup specific studies and appropriate validation in human disease.

## 6 Limitations and future research directions

Several limitations to the current understanding of MB-TME are worthwhile to highlight. While the MB-TME is being studied through novel omics-based technologies, published studies and datasets remain few due to the high financial cost, high computational intensity, and multidisciplinary expertise required. Bulk RNA sequencing technologies captures the aggregate gene expression across tissue and therefore do not directly capture immune heterogeneity or spatial localization of gene signal. Transcriptomics has uncovered significant insight to the MB-TME composition and cell states, however large-scale studies of non-malignant components of the MB-TME remain limited, particularly in endothelial, neural, and glial populations. Many spatial platforms are constrained by spatial resolution and therefore show blended gene expression from a spatial spot where gene expression from rare cell types may be lost. Visium supports standard resolution sequencing with 55mm spots which may allow up to 10-cells per voxel (98), though emerging technologies are mitigating this limitation. In 2024, a high-definition spatial platform was released with 2mm resolution. Currently, sub-cell spatial resolution can be achieved through imaging-based platforms such as Xenium, however, whole-transcriptome coverage is lost.

Another challenge for investigation and validation of the functional roles of the MB-TME is the limited availability of appropriate disease models, therefore necessitating either fresh surgical specimen or formalin-fixed paraffin-embedded tissue. However, this snapshot of the TME at one period of time does not address the evolution of the TME in response to treatment and as the tumor progresses. Appropriate models of disease that recapitulate the TME with spatial fidelity would eliminate this barrier to study, however, to date no such models exist. Murine models are limited by cross-species difference in biology. PDX models contain a cross-species host which further introduces differences to TME biology as the tumor may be infiltrated by murine host immune cells. Organoids represent a new frontier for human disease modeling and may lower the barrier to studying MB-TME, but validation of these models is needed to accurately reflect disease pathogenesis and predict treatment efficacy and outcomes. Likewise, introduction of TME components to the organoid models remains a challenge. Further complicating our understanding of the precise role of the TME in MB is the small patient cohort sizes in which investigation has taken place, the further subtyping of the major molecular subgroups into smaller components.

Collectively, these limitations require an appropriate degree of caution in their interpretation. Mechanistic insights inferred through bioinformatics may strengthen with orthogonal validation in preclinical disease modeling, and results of preclinical disease models should still be carefully studied in translation to human disease.

Despite these limitations, recent technological advancements may allow for the discovery of novel insights. The recent introduction of spatial proteomic platforms expands upon prior methods by retaining the protein spatial positioning and was highlighted by *Nature Methods* as the “2024 Method of the Year” (99). Overall, a more thorough characterization of the MB-TME may reveal novel molecular and cellular interactions and signaling crosstalk that could influence disease initiation, proliferation, metastasis, or treatment resistance. Future studies focused on understanding exactly how the TME modulates the pathogenesis of MB may uncover innovative new therapeutic approaches for the treatment of MB. An integrated multi-omic approach with data from genomics, transcriptomics, proteomics, and lipidomic sources utilizing blood, CSF, and tissue from human MB patients can provide more comprehensive insight into TME biology. Such an approach will mitigate transcriptome-protein discordance and help to validate the preclinical murine models for understanding the mechanistic interactions between tumor-TME that can be exploited therapeutically.

Finally, potential AI-based approaches to understanding the MB-TME include radiomic feature extraction or digital pathology analysis with early success in other neuro-oncologic histologies. Radiomics may provide a less invasive approach to risk stratification or molecular characterization. Deep learning-based strategies trained on digitized pathology may reveal novel patterns of spatial architecture and further may orthogonally test conclusions drawn from the transcriptomics scale. The application of AI to neuro-oncology may be a mechanism for elucidating the evolving composition and interactions of the TME in real time through the course of treatment, thus tailoring our understanding of the TME on an individual patient basis. Should this be realized, personalized treatment strategies could conceivably be developed that focus on disrupting the key interactions between tumor cells and the constituents of the local environment that are promoting patient-specific tumor progression.
